# Comparative Performance Analysis of Lightweight Cryptographic Algorithms on Resource-Constrained IoT Platforms †

**DOI:** 10.3390/s25185887

**Published:** 2025-09-20

**Authors:** Tiberius-George Sorescu, Vlad-Mihai Chiriac, Mario-Alexandru Stoica, Ciprian-Romeo Comsa, Iustin-Gabriel Soroaga, Alexandru Contac

**Affiliations:** 1 Department of Telecommunications, “Gheorghe Asachi” Technical University of Iași, 700506 Iași, Romania; vlad-mihai.chiriac@academic.tuiasi.ro (V.-M.C.); mario-alexandru.stoica@student.tuiasi.ro (M.-A.S.); ciprian-romeo.comsa@academic.tuiasi.ro (C.-R.C.); iustin-gabriel.soroaga@student.tuiasi.ro (I.-G.S.); 2 Department of Computer Science, “Gheorghe Asachi” Technical University of Iași, 700050 Iași, Romania; alexandru.contac@student.tuiasi.ro

**Keywords:** IoT security, lightweight cryptography, stream ciphers, energy efficiency, performance analysis, Nordic Thingy:53, bluetooth mesh

## Abstract

The increase in Internet of Things (IoT) devices has introduced significant security challenges, primarily due to their inherent constraints in computational power, memory, and energy. This study provides a comparative performance analysis of selected modern cryptographic algorithms on a resource-constrained IoT platform, the Nordic Thingy:53. We evaluated a set of ciphers including the NIST lightweight standard ASCON, eSTREAM finalists Salsa20, Rabbit, Sosemanuk, HC-256, and the extended-nonce variant XChaCha20. Using a dual test-bench methodology, we measured energy consumption and performance under two distinct scenarios: a low-data-rate Bluetooth mesh network and a high-throughput bulk data transfer. The results reveal significant performance variations among the algorithms. In high-throughput tests, ciphers like XChaCha20, Salsa20, and ASCON32 demonstrated superior speed, while HC-256 proved impractically slow for large payloads. The Bluetooth mesh experiments quantified the direct relationship between network activity and power draw, underscoring the critical impact of cryptographic choice on battery life. These findings offer an empirical basis for selecting appropriate cryptographic solutions that balance security, energy efficiency, and performance requirements for real-world IoT applications.

## 1. Introduction

The Internet of Things (IoT) has exceeded 16 billion active devices as of 2025 [1], creating unprecedented security challenges due to inherent resource constraints in computational power, memory, and energy [2,3].

Existing cryptographic performance evaluations exhibit critical limitations that constrain their applicability to modern IoT deployments. Potlapally et al. [4,5] established energy measurement methodologies using legacy PDA platforms that poorly represent contemporary IoT architectures. While Aslan et al. [6] evaluated lightweight algorithms on MSP430 platforms, their analysis excluded network operation impacts that dominate real-world energy budgets. Similarly, Radosavljević and Babić [7] developed mathematical models but lacked empirical validation on physical hardware under realistic operating conditions.

This research synthesis reveals a critical gap: no existing study provides comprehensive energy consumption analysis of NIST-selected lightweight cryptographic standards alongside established eSTREAM finalists under realistic Bluetooth mesh networking conditions using contemporary IoT hardware platforms. Our work directly addresses this void through dual-scenario empirical evaluation that captures both network protocol overhead and computational efficiency trade-offs.

This study addresses how modern lightweight cryptographic algorithms perform in terms of energy efficiency under realistic Bluetooth mesh networking conditions, what performance trade-offs exist between AEAD-integrated algorithms and traditional stream ciphers requiring separate MAC computation, and how algorithm selection decisions impact battery life in resource-constrained IoT deployments.

Our contributions include the first dual-scenario evaluation of ASCON alongside eSTREAM finalists on modern IoT hardware, quantitative battery life projections for realistic deployment scenarios, and an empirical framework for algorithm selection based on application-specific requirements.

### Platform and Algorithm Selection Rationale

The selection of the Nordic Thingy:53 platform was based on several key factors that make it representative of contemporary IoT devices. Built around the nRF5340 dual-core ARM Cortex-M33 SoC, this platform provides realistic constraints typical of modern IoT edge devices while offering comprehensive support for Bluetooth mesh networking [8]. The dual-core architecture enables efficient task separation, with one core dedicated to application processing while the other handles radio communications, making it ideal for evaluating cryptographic implementations in networked scenarios [9,10].

The exclusion of AES from direct performance comparison was necessitated by the Nordic Thingy:53 platform’s dedicated hardware acceleration for AES operations. Including AES would have unfairly skewed the evaluation, as its hardware-optimized implementation contrasts with the purely software-based nature of the other algorithms tested. While this limitation may affect the generalizability of results to platforms without hardware-accelerated AES, it ensures that our comparative analysis focuses on the intrinsic computational efficiency of the evaluated algorithms under equivalent conditions. The findings remain applicable to the broad category of resource-constrained IoT devices that rely on software-implemented cryptographic solutions [11].

The next section, Section 2 presents a detailed survey of the cryptographic algorithm designs under review. We then transition to Section 3 and Section 4, our methodology and experimental setup, where we detail the Bluetooth mesh experimental setup on the Nordic Thingy:53 hardware platform. Section 5 presents the core of our findings, where we analyze the energy consumption and security trade-offs of each cipher and provide an in-depth interpretation of performance anomalies, such as the significant power spikes observed during HC-256 operations. Finally, Section 6 consolidates our results and discussion into a set of concluding remarks on the practical implications for securing IoT ecosystems.

## 2. Cipher Algorithms

### 2.1. XChaCha20: Extended-Nonce Stream Cipher

ChaCha20 is a 20-round stream cipher that takes a 256-bit key, a 32-bit little-endian block counter, and a 96-bit nonce, producing 64-byte keystream blocks via an add-rotate-xor “quarter-round” function [12]. Using a nonce of 96-bit length, the ChaCha20 is able to encrypt $\approx 2^{48}$ msgs/key [12]. In systems with high throughput or asynchronous delivery, managing a limited nonce space risks collisions. XChaCha20 solves this problem by accepting 192-bit nonce, reducing the collision probability to negligible levels in practice [13]. XChaCha20 keeps the same core but feeds a 24-byte (192-bit) nonce into a single “HChaCha20” block: the first 16 nonce bytes and the key generate a sub-key, while the remaining 8 bytes (prefixed with four zero counter bytes) become the normal ChaCha20 nonce, so encryption speed is identical after this one-block setup. The extended nonce raises the random-nonce collision bound from $2^{48}$ to $2^{96}$ messages per key, making it safer for long-lived keys and high-volume systems.

### 2.2. HC-256: High-Performance Stream Cipher

HC-256, designed by Hongjun Wu and published in 2004, was a finalist in the eSTREAM software profile. It aims for bulk encryption at high throughput while maintaining strong confidence in security [14]. HC-256 expands two 1024-entrytables via non-linear feedback and AES-inspired S-box look-ups [15]. Its 4 KB internal state ensures high diffusion but taxes caches on embedded MCUs. The security architecture of HC-256 is based on the difficulty of inverting the algorithm’s non-linear update functions and the large internal state size of 65,536 bits [14]. This substantial state space provides security against various cryptanalytic attacks, including distinguishing attacks and state recovery attempts. However, the algorithm’s memory requirements present challenges for implementation on highly resource-constrained devices [11].

Performance evaluations demonstrate that HC-256 achieves excellent throughput on platforms with sufficient memory resources. The algorithm’s design specifically targets modern processor architectures with large caches, making it well suited for applications where memory is not the primary constraint [11].

### 2.3. Sosemanuk: Software-Oriented Stream Cipher

Sosemanuk represents a sophisticated fusion of design principles from the SNOW 2.0 stream cipher and the Serpent block cipher, optimized specifically for software implementation [16]. The algorithm supports variable key lengths from 128 to 256 bits and utilizes a 128-bit initialization vector, claiming 128-bit security regardless of key length.

The cryptographic architecture of Sosemanuk consists of a linear feedback shift register (LFSR) component derived from SNOW 2.0 and a finite state machine incorporating Serpent S-boxes [16]. This hybrid approach aims to combine the efficiency of Linear Feedback Shift Register (LFSR)-based designs with the non-linear strength provided by well-analyzed block cipher components.

Recent cryptanalytic research has revealed potential vulnerabilities in Sosemanuk’s security margin, with improved fast correlation attacks demonstrating state recovery with time complexity of $2^{134.8}$ [17], significantly faster than previously achieved. These findings suggest that while Sosemanuk maintains practical security for current applications, its security margin is approximately 28 bits for 128-bit security, indicating potential concerns for long-term cryptographic applications. Thus, designers and system architects should approach the adoption of the Sosemanuk algorithm with caution in industrial IoT deployments where software updates are impractical and hardware modifications are challenging.

### 2.4. ASCON

Ascon is a family of algorithms providing authenticated encryption with associated data (AEAD) and hashing functionalities [18,19]. In 2023, the National Institute of Standards and Technology (NIST) selected Ascon as the new standard for lightweight cryptography, making it suitable for resource-constrained devices like those used in the Internet of Things (IoT) [18,20]. Its design aims to ensure confidentiality, integrity, and authenticity for such applications. The algorithm is built on a sponge-like framework that uses a 320-bit internal state [21]. This state is partitioned into five 64-bit words, delivering a 128-bit security level [21]. The core of Ascon’s operation is a permutation function known as Ascon-p, which is applied in a series of rounds to process data. The number of rounds varies depending on the specific operation being performed. The process involves several distinct stages:

- Initialization: The internal state is set up using a secret key, a unique nonce, and an initial value. This is followed by a full 12-round permutation.
- Processing Associated Data: Any associated data, such as headers or protocol information, are absorbed into the state, followed by a 6-round permutation.
- Encryption: The plaintext is processed similarly to the associated data, also using a 6-round permutation for each block.
- Finalization: The process concludes with another 12-round permutation, after which a 128-bit authentication tag is generated. This tag depends on the entire history of the operation, including the nonce, associated data, and ciphertext, ensuring that any modification will be detected during verification.

The Ascon-p permutation consists of three repeating steps in each round: adding a round constant, applying a non-linear substitution layer, and applying a linear diffusion layer. These steps are designed to be efficiently implemented in both hardware and software [22].

### 2.5. Rabbit

Another efficient stream cipher is Rabbit, developed by M. Boesgaard et al. [23]. Rabbit, which employs a 128-bit secret key and a 64-bit initialization vector (IV), showed very good efficiencies in encrypting and decrypting data. The algorithm uses inexpensive word-level operations—32-bit modular addition, XOR, and fixed rotations—facilitating constant-time implementations free of table look-ups or S-boxes. These efficiency and implementation advantages earned Rabbit a place in the final eSTREAM software portfolio [24].

The strength of the algorithm comes from the mixing of eight counters with eight internal states and a single carry bit using arithmetical operations. Each clock step refreshes the counters with key-dependent constants, feeds the resulting sums through a non-linear squaring operation, and mixes the outputs with rotations and additions. A full diffusion across the 256-bit state is provided by the algorithm in just two cycles.

### 2.6. Salsa20

Salsa20, designed by D. J. Bernstein, was selected as a finalist in the eSTREAM project because of its high throughput and simple software implementation [25]. As a stream cipher, it produces a pseudorandom keystream that is XOR-ed with plaintext bytes to yield ciphertext and vice-versa. Keystream generation is driven by a 256-bit (optionally 128-bit) secret key, a 64-bit nonce, and a 64-bit block counter. Internally, Salsa20 relies on three word-level operations—32-bit modular addition, 32-bit XOR, and fixed-distance 32-bit left rotation—combined in a quarter-round transformation that provides rapid diffusion.

During the eSTREAM project, three versions of the algorithm were tested: Salsa20/20, Salsa20/12, and Salsa20/8, comprising 20, 12, and 8 full rounds, respectively [24]. The 20-round variant remains the conservative choice for maximum security, while the 12-round version achieves a widely accepted balance between security margin and performance, especially on resource-constrained platforms.

In operation, Salsa20 first packs 16 little-endian 32-bit words—four fixed ASCII constants, eight key words, a 64-bit block counter, and a 64-bit nonce—into a 4 × 4 state matrix. Each full “round” consists of four column quarter-rounds followed by four row quarter-rounds. Every quarter-round combines its four input words with one add–rotate–xor (ARX) sequence, rapidly mixing bits across the matrix. The algorithm adds the final state to the original input state (“feed-forward”), then serializes the words to produce 64 bytes of keystream, ready to be XOR-ed with the corresponding plaintext block. This simple ARX core avoids S-boxes and table look-ups, yielding constant-time software that is both fast on general-purpose CPUs and resistant to cache-timing attacks.

### 2.7. Similarities and Differences

All the ciphers presented are symmetric cryptographic algorithms designed for fast and secure data encryption. While they share the common goal of generating a pseudorandom keystream to be combined with plaintext, they differ in their design, performance characteristics, and ideal use cases. Most of these algorithms function as stream ciphers. The fundamental principle of a stream cipher is to generate a long sequence of pseudorandom bits, known as the keystream, from a secret key and a unique value called a nonce or Initialization Vector (IV) [26]. The primary differences lie in their internal structure, key and nonce sizes, performance, and the security features they offer. These ciphers emerged from different cryptographic research initiatives, notably the eSTREAM project for high-performance stream ciphers and the NIST Lightweight Cryptography (LWC) competition for efficient and secure algorithms in constrained environments. ASCON stands apart from the others as the winner of the NIST Lightweight Cryptography (LWC) competition. Its primary goal is to provide security for resource-constrained devices like IoT sensors and microcontrollers. The most significant difference is that ASCON is an Authenticated Encryption with Associated Data (AEAD) cipher. This means it provides not only confidentiality (encryption) but also integrity and authenticity in a single, integrated operation. The other ciphers on this list are purely stream ciphers and require a separate Message Authentication Code (MAC) algorithm to provide the same level of protection. Salsa20 and XChaCha20 (a 192-bit-nonce extension) share the same Add-Rotate-XOR round function, giving them similar software performance profiles. Every algorithm keeps its internal state in 32-bit words, which maps well to 32-bit micro-controllers common in IoT nodes. All six require a fresh, unpredictable nonce/IV for every message to avoid keystream reuse. Each design aims at a small memory footprint (<4 kB RAM, few kilobytes of code).

Table 1 summarizes the key characteristics of the evaluated stream ciphers, including security parameters, nonce/IV requirements, and implementation aspects. This comparison provides a concise overview to guide algorithm selection for resource-constrained IoT environments.

## 3. Methodology

This study employs a dual-testbench methodology designed to evaluate cryptographic algorithms under two distinct operational paradigms representative of real-world IoT deployments. The experimental design addresses the fundamental question of how cryptographic algorithm selection affects both computational efficiency and energy consumption in resource-constrained networked environments [27,28].

### 3.1. Experimental Design Overview

The methodology addresses potential limitations in current IoT cryptographic evaluation approaches by implementing controlled experiments that isolate the effects of algorithm choice from other variables. Two complementary testing scenarios were designed: (1) Bluetooth mesh networking evaluation to assess algorithm performance under realistic IoT communication patterns [29,30], and (2) high-throughput bulk data transfer testing to evaluate computational and energy overhead during intensive cryptographic operations.

### 3.2. Experimental Limitations and Mitigation Strategies

Several methodological limitations were acknowledged and addressed in the experimental design. The reuse of identical keys and nonces across experiments, while violating security best practices, was necessary to ensure measurement repeatability and eliminate variables that could confound energy consumption analysis. This approach allows direct comparison of algorithmic computational costs while acknowledging that real-world deployments would require proper key management protocols [2,3].

The linear topology employed in Bluetooth mesh testing represents a simplified network structure compared to complex, dynamic mesh topologies encountered in practical deployments [29,30]. However, this controlled approach enables isolation of cryptographic effects from topology-dependent variables, such as routing overhead and network congestion. Future work should examine algorithm performance under more complex network topologies to validate the generalizability of these findings.

### 3.3. Acknowledged Methodological Constraints

The experimental design incorporates several controlled simplifications that require careful interpretation of results. Identical keys and nonces were used across experimental trials to enable direct algorithmic comparison while eliminating cryptographic setup variables. This approach violates security best practices but provides conservative energy estimates, as real deployments would incur additional key management overhead. The results represent computational efficiency baselines rather than complete deployment costs.

The linear Bluetooth mesh topology enables controlled isolation of cryptographic effects while eliminating topology-dependent variables, such as routing complexity and network congestion. This simplified network structure provides lower-bound energy consumption estimates, as complex mesh topologies would proportionally increase routing overhead across all evaluated algorithms. The controlled approach allows direct comparison of cryptographic computational costs without confounding variables from dynamic network behavior.

Evaluation on the Nordic Thingy:53 platform provides detailed baseline measurements for ARM Cortex-M33 architectures, though platform-specific optimizations may not transfer directly to other IoT hardware. The findings establish performance rankings and relative efficiency relationships that inform algorithm selection within similar resource-constrained environments, while requiring validation studies on diverse architectures for broader generalizability.

### 3.4. Bluetooth Mesh Technology

Bluetooth mesh networking has emerged as a critical technology for large-scale IoT deployments, enabling scalable, self-healing networks of interconnected devices [27,28]. Bluetooth mesh networking extends the capabilities of traditional Bluetooth Low Energy by enabling many-to-many device communications over a mesh topology. The technology’s flooding-based message propagation mechanism ensures robust communication while introducing unique energy consumption patterns that vary significantly based on cryptographic implementations [29,30].

## 4. Experimental Setup

### 4.1. Hardware Platform Specifications

The Nordic Thingy:53 development kit features a dual-core ARM Cortex-M33 architecture specifically optimized for low-power IoT applications [9]. The nRF5340 SoC includes dedicated hardware acceleration for AES operations, which necessitated its exclusion from comparative analysis to ensure fair evaluation of software-implemented algorithms. The platform’s power profiling capabilities provide precise energy consumption measurements during cryptographic operations with ±0.1mA accuracy [8].

This platform has been successfully utilized in various machine learning and sensor applications, demonstrating its capability for real-time data processing with minimal energy consumption [10]. The dual-core design enables efficient task separation, with one core dedicated to application processing while the other handles radio communications, making it ideal for Bluetooth mesh implementations [27,28].

### 4.2. Experimental Workflow Methodologies

This section presents the detailed workflow diagrams for our dual-testbench methodology, illustrating the systematic processes employed in both bulk data transfer evaluation and Bluetooth mesh network testing. These workflows ensure reproducible measurements and isolate the effects of cryptographic algorithm selection on energy consumption patterns.

#### 4.2.1. High-Throughput Bulk Data Transfer Workflow

**Figure 1:**
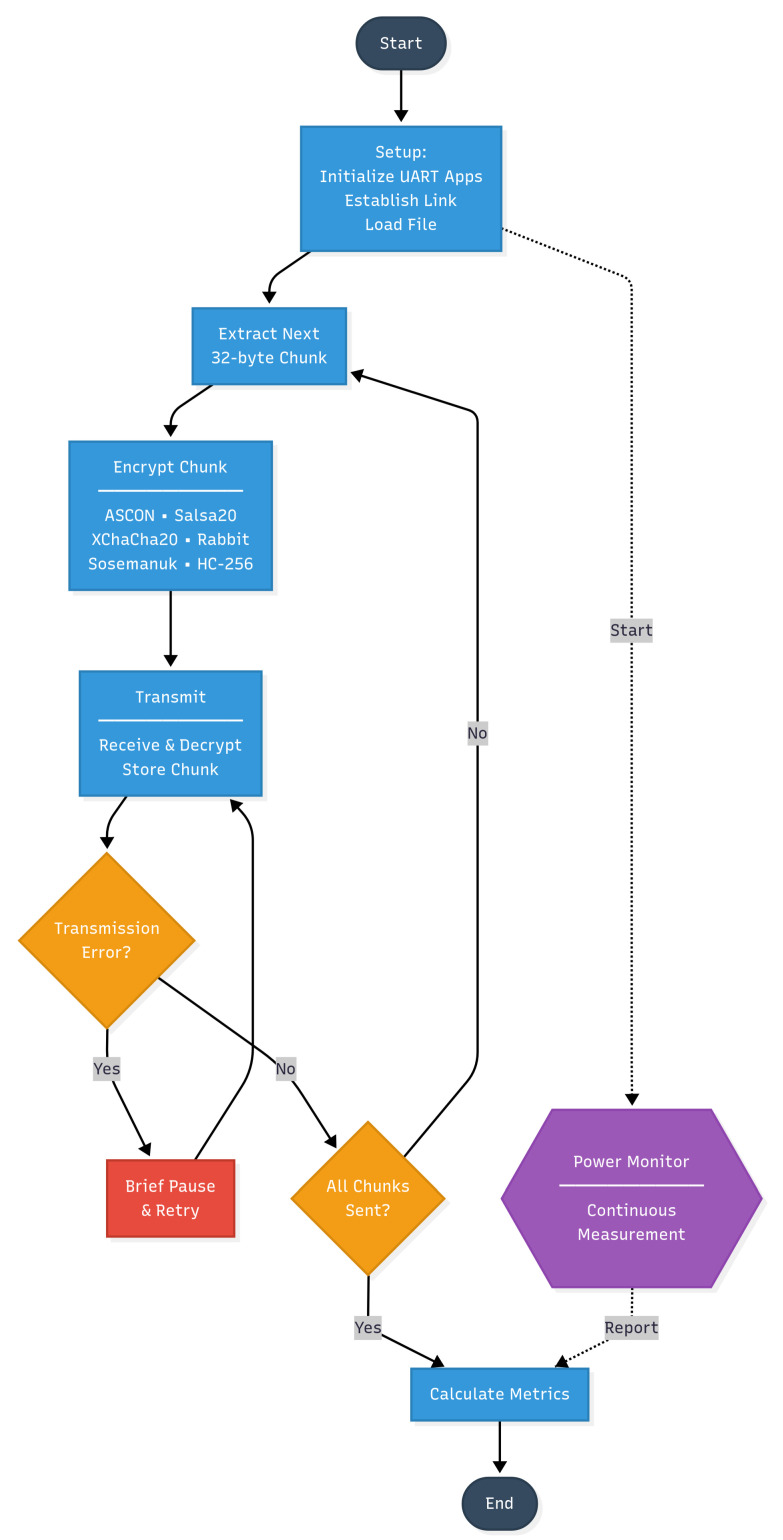
High-throughput bulk data transfer workflow showing the systematic process for evaluating cryptographic algorithms during intensive data encryption scenarios.

Figure 1 illustrates the systematic process employed for evaluating cryptographic algorithms during high-throughput bulk data encryption scenarios. The workflow begins with a comprehensive setup phase where UART applications are initialized, communication links are established between devices, and data files are loaded into memory. This initial phase ensures consistent starting conditions across all algorithmic evaluations.

The core processing loop extracts sequential 32-byte chunks from the source data, representing typical IoT data packet sizes encountered in firmware updates and sensor data aggregation scenarios. Each chunk undergoes encryption using one of the six evaluated algorithms: ASCON (both 32-bit and 64-bit variants), Salsa20, XChaCha20, Rabbit, Sosemanuk, and HC-256. The encrypted chunks are then transmitted via Bluetooth communication, received by the destination device, decrypted, and stored for integrity verification.

Transmission errors trigger brief pause-and-retry sequences, preventing network congestion from affecting cryptographic performance measurements.

Continuous power monitoring runs parallel to the main processing workflow, capturing detailed energy consumption profiles throughout the encryption, transmission, and processing phases.

#### 4.2.2. Bluetooth Mesh Network Temperature Monitoring Workflow

**Figure 2:**
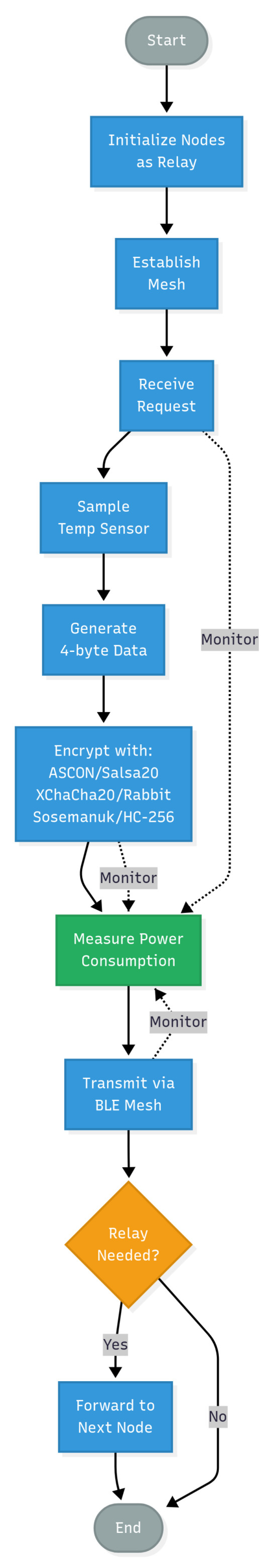
Bluetooth mesh network temperature monitoring workflow demonstrating the systematic process for evaluating cryptographic algorithms in realistic IoT sensor deployment scenarios.

Figure 2 presents the workflow for Bluetooth mesh networking evaluation, simulating realistic IoT sensor deployment scenarios. The process initiates with mesh network establishment, where multiple Nordic Thingy:53 devices are configured as relay nodes to create a multi-hop communication topology. This setup reflects real-world IoT deployments where sensor data must traverse multiple network hops to reach collection points.

Upon receiving data requests, each node samples its onboard temperature sensor and generates 4-byte data packets containing temperature readings. The generated data undergo encryption using the same six cryptographic algorithms evaluated in the bulk transfer scenario.

Power consumption measurement occurs continuously throughout the encryption and transmission phases, capturing the energy cost of both cryptographic operations and mesh networking protocols. The encrypted sensor data are transmitted via Bluetooth mesh protocols, with intermediate nodes performing relay operations as required by the network topology.

The workflow includes decision logic for relay forwarding, where intermediate nodes determine whether received packets require forwarding to maintain network connectivity. This multi-hop communication pattern reflects the energy costs of mesh networking in battery-powered sensor deployments.

## 5. Results

The experimental evaluation conducted on the Nordic Thingy:53 development board provides performance metrics for both standalone cryptographic operations and integrated Bluetooth mesh networking scenarios. The results demonstrate significant variations in computational efficiency and power consumption across different cipher implementations. The algorithms have been sorted alphabetically, and the results are presented in two main sections: the first focuses on the performance of each cipher in a Bluetooth mesh network context, while the second section evaluates their performance during high-throughput data transfers.

### 5.1. Statistical Analysis and Reproducibility

The initial study [31] concentrated on a select group of ciphers (ASCON, Salsa20, and XChaCha20) and their power consumption within Bluetooth mesh networks, while the current work adds three more stream ciphers and employs a dual-benchmark experimental approach that covers both Bluetooth mesh networking and high-throughput bulk data transfer scenarios.

To assess the consistency and significance of the performance differences between cryptographic algorithms, we conducted a basic statistical analysis using the raw measurement data collected during both test scenarios.

For each algorithm, we performed 10 repeated trials to measure average power consumption and throughput under both the Bluetooth mesh and high-throughput transfer benchmarks.

These findings confirm that the performance rankings observed are not due to random variation and that the choice of cryptographic algorithm has a statistically meaningful impact on energy efficiency and throughput in constrained IoT scenarios.

### 5.2. Bluetooth Mesh Network Performance Analysis

**Figure 3:**
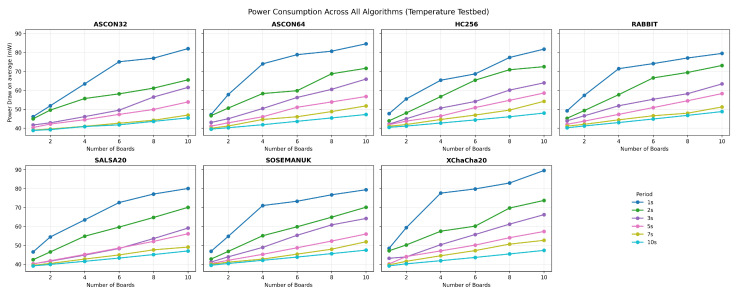
Power consumption comparison across all cryptographic algorithms in Bluetooth mesh networks. Each panel shows performance scaling from 2 to 10 boards with varying communication periods (1 s to 10 s intervals).

The mesh network evaluation reveals distinct power consumption patterns across communication frequencies, as illustrated in Figure 3, which consolidates the performance characteristics of all seven evaluated algorithms. The multi-panel visualization demonstrates how power consumption scales with both network size (2–10 boards) and reporting frequency (1 s to 10 s intervals).

**Figure 4:**
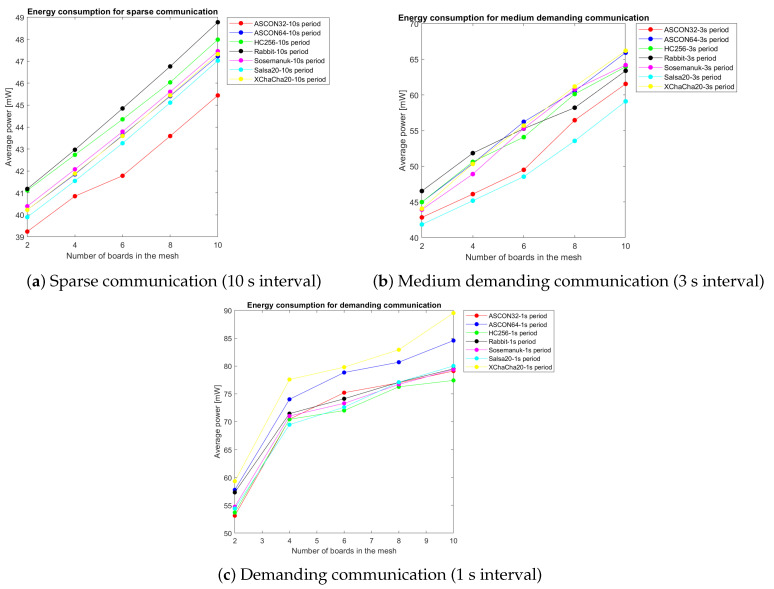
Power consumption comparison across all ciphers under different communication scenarios: (**a**) sparse communication with 10 s reporting interval, (**b**) medium demanding communication with 3 s reporting interval, and (**c**) demanding communication with 1 s reporting interval.

Figure 4 summarizes the power consumption comparison across communication scenarios.

Three distinct operational patterns emerge from the mesh network analysis:

- Sparse Communication (10 s intervals)
- Medium-Demanding Communication (3 s intervals)
- Demanding Communication (1 s intervals)

Figure 4a demonstrates minimal algorithmic differentiation, with all ciphers converging to baseline power consumption levels between 46–50 mW. This scenario represents typical sensor network deployments where the cryptographic overhead becomes negligible relative to the radio idle time.

Figure 4b reveals algorithm-specific performance characteristics becoming more pronounced. Salsa20 demonstrates optimal efficiency at 58.4 mW, while XChaCha20 shows 13% higher consumption due to extended nonce processing overhead.

Figure 4c exposes maximum performance differentiation. Sosemanuk achieves the lowest consumption at 79.8 mW, while XChaCha20 peaks at 89.2 mW—an 11.8% difference that becomes critical for battery-constrained deployments.

### 5.3. High-Throughput Bulk Transfer Performance

**Figure 5:**
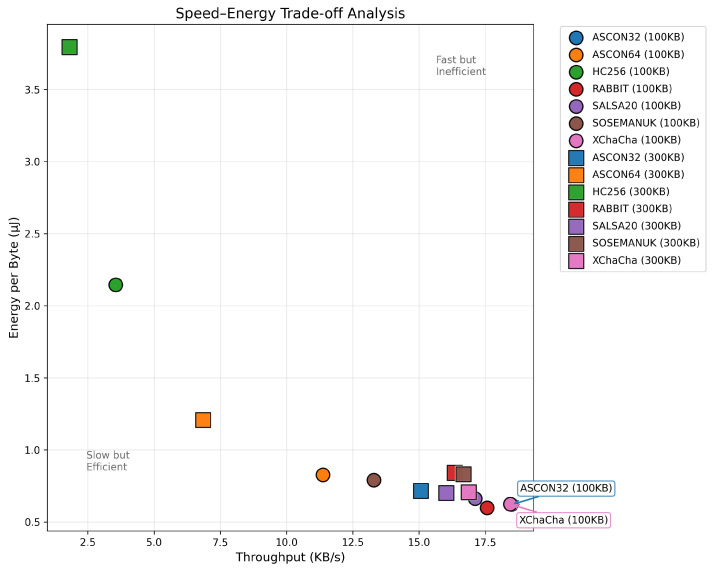
Speed–energy trade-off analysis for bulk transfers. The optimal region (bottom-right) indicates high throughput with low energy consumption.

The bulk transfer evaluation using 300KB payloads reveals dramatically different performance characteristics compared to mesh networking scenarios. The speed–energy trade-off analysis is presented in Figure 5. ASCON32, XChaCha20 and Salsa20 occupy the optimal corner with high throughput (>16 KB/s) and low energy consumption (<0.75 µJ/byte), while HC-256 represents a clear outlier with both poor throughput and high energy consumption.

### 5.4. Practical Battery Life Projections for IoT Deployments

To translate our experimental findings into practical deployment guidance, Table 2 presents battery life projections for the Nordic Thingy:53 (1500 mAh battery at 3.7 V, 5550 mWh total energy) under representative IoT operational scenarios. These calculations assume continuous operation with the specified cryptographic algorithm and do not account for sleep modes or duty cycling commonly employed in production deployments.

#### Algorithm Selection Rationale

For continuous mesh networking applications, such as environmental monitoring, the choice of cryptographic algorithm significantly impacts operational lifetime. ASCON32 emerges as the optimal choice for sparse communication patterns with intervals of 10 seconds or longer, providing the longest battery life while offering integrated AEAD functionality that eliminates the need for separate MAC computation. This dual benefit of energy efficiency and simplified security implementation makes it particularly attractive for resource-constrained deployments.

In contrast, when dealing with high-frequency communication scenarios requiring 1-second intervals, Sosemanuk demonstrates superior efficiency despite the security margin concerns discussed in Section 2.3. For deployments that can implement regular key rotation protocols, Sosemanuk remains a viable option, achieving approximately 69.5 h of continuous operation compared to other algorithms that consume more power under these demanding conditions. Notably, XChaCha20 should be avoided in continuous mesh operations as it exhibits 11.8% higher power consumption compared to the most efficient alternatives, which translates to approximately 7–9 h of reduced operational time per charge cycle.

The dynamics change dramatically for bulk transfer operations such as firmware updates, where the energy impact is determined by transfer duration rather than steady-state power consumption. XChaCha20 paradoxically becomes the preferred choice in these scenarios despite its higher instantaneous power draw, as it completes 1 MB transfers in just 54.2 s compared to HC-256’s impractical 281.4 s. The total energy consumed, calculated as the product of duration and power, strongly favors faster algorithms even when their instantaneous power consumption is higher. HC-256 proves particularly unsuitable for bulk operations, consuming 5.4 times more energy than XChaCha20 for identical payloads due to its extended processing time, making it a poor choice for battery-powered devices requiring periodic firmware updates.

### 5.5. Algorithm Selection Framework

**Figure 6:**
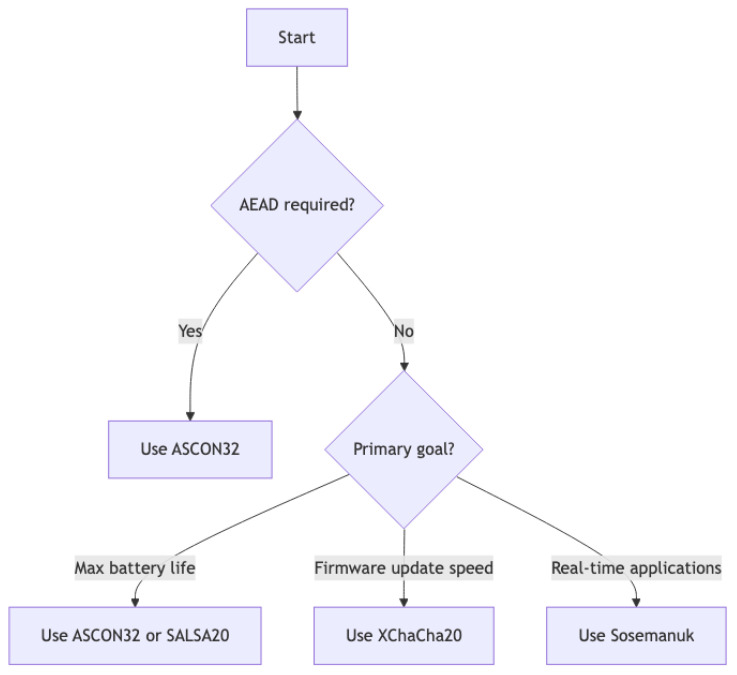
Cryptographic algorithm selection framework based on deployment requirements.

Figure 6 presents a practical decision framework for algorithm selection in IoT deployments. The framework yields the following recommendations:

- AEAD requirement: ASCON32 provides authenticated encryption with competitive performance
- Battery optimization: ASCON32 or Salsa20 for balanced mesh/bulk performance (107.4 and 104.7 h, respectively, at 10 s intervals)
- Firmware updates: XChaCha20 for minimal transfer time (17.78 s for 300 KB)
- Real-time applications: Sosemanuk for lowest mesh power (62.0 h at 1 s intervals), with security trade-offs considered

### 5.6. Threat Model for IoT Cryptographic Implementations

IoT devices face a multi-faceted threat landscape that extends beyond traditional cryptanalytic attacks. Physical access to devices enables side-channel attacks, including power analysis, electromagnetic emanation analysis, and fault injection attacks [2,3]. The algorithms evaluated in this study exhibit varying resilience to such attacks.

Stream ciphers with regular operation patterns (Salsa20, XChaCha20) may be more susceptible to timing and power analysis compared to algorithms with irregular state updates [12,25]. ASCON’s permutation-based design provides some inherent resistance to differential power analysis, though dedicated countermeasures would be required for high-security applications [18,19,20]. HC-256’s large internal state and complex operations may provide natural resistance to simple power analysis but could be vulnerable to more sophisticated attacks [11,14].

### 5.7. Comparison with AES Hardware Acceleration

While excluded from direct performance comparison due to hardware acceleration, AES represents the most widely deployed cryptographic standard in IoT devices. The Nordic nRF5340’s dedicated AES coprocessor enables encryption rates exceeding 1 MB/s while consuming approximately 3–5 mW during operation [8]. However, this performance advantage comes with trade-offs: hardware AES implementations may be more vulnerable to fault injection attacks, and the fixed functionality limits algorithmic agility [2,3].

In scenarios where hardware AES is available, the choice between hardware-accelerated AES and software-implemented alternatives depends on specific application requirements. For applications requiring AEAD functionality, hardware AES would necessitate additional MAC computation, potentially negating its performance advantage compared to ASCON’s integrated approach [18,19].

## 6. Conclusions and Future Directions

This study provides empirical evidence for cryptographic algorithm selection specifically targeting Nordic nRF5340-based platforms operating in Bluetooth mesh networks. The controlled evaluation demonstrates algorithm-specific energy consumption differences of up to 11.8% in network scenarios and substantial variations in bulk transfer performance, establishing quantitative baselines for deployment decision-making.

### 6.1. Platform-Specific Findings and Implementation Guidance

Our evaluation on ARM Cortex-M33 architectures demonstrates that ASCON32 achieves optimal energy efficiency for sparse communication scenarios while providing integrated AEAD functionality that eliminates separate MAC computation requirements. This dual benefit of energy efficiency and simplified security implementation makes it particularly attractive for resource-constrained deployments where battery life optimization is critical. In contrast, XChaCha20 minimizes bulk transfer duration through superior computational efficiency but increases mesh networking overhead compared to optimal alternatives, making it suitable for firmware update scenarios where transfer speed prioritizes over steady-state power consumption.

The findings reveal that HC-256 exhibits prohibitive energy consumption for both networking and bulk transfer scenarios, consuming substantially more power than alternatives across all tested configurations. Algorithm selection significantly impacts battery life projections in continuous operation scenarios, with differences translating to measurable operational time variations that affect deployment viability in battery-powered systems.

### 6.2. Research Extensions and Practical Applications

For Nordic nRF5340-based deployments, the evidence supports algorithm selection based on specific operational requirements rather than generic security considerations alone. Environmental monitoring applications benefit from ASCON32’s energy efficiency and integrated authentication capabilities, while firmware update scenarios favor XChaCha20 for minimized transfer duration despite higher instantaneous power consumption. High-frequency sensing applications may consider Sosemanuk with appropriate evaluation of security margin implications given recent cryptanalytic developments.

Future research priorities include platform diversity validation across RISC-V and alternative ARM architectures to establish broader hardware applicability, complex mesh topology evaluation under realistic node density and mobility patterns that reflect practical deployment conditions, and statistical rigor enhancement through formal significance testing and confidence interval analysis to strengthen empirical foundations. Cross-protocol validation extending to LoRaWAN and Zigbee networking stacks would further expand the applicability of these findings to diverse IoT deployment scenarios.

These findings establish a validated foundation for cryptographic algorithm selection within the evaluated platform constraints while acknowledging the need for extended validation studies before broader generalization across the diverse landscape of IoT hardware and networking technologies.

## Figures and Tables

**Table 1:** Key characteristics of evaluated stream ciphers.

Feature	Salsa20	XChaCha20	SOSEMANUK	Rabbit	HC-256	ASCON
Origin	eSTREAM Winner	IETF Standard	eSTREAM Finalist	eSTREAM Finalist	eSTREAM Candidate	NIST LWC Winner
Design Principle	Add-Rotate-XOR (ARX)	ARX, extended nonce	SNOW/SERPENT-based	Custom	Large state tables	Permutation-based sponge
Key Size (bits)	128 or 256	256	128 to 256	128	256	128
Nonce/IV Size (bits)	64	192	128	64	256	128
Internal State (bits)	512	512	384	∼513	65,536 (for HC-256)	320
Authenticated Encryption	No (requires separate MAC)	No (requires separate MAC)	No (requires separate MAC)	No (requires separate MAC)	No (requires separate MAC)	Yes (Integrated)

**Table 2:** Battery life projections and algorithm recommendations.

Scenario/Algorithm	Power Draw	Battery Life	Recommendation
*Mesh network—Environmental monitoring*			
Sparse (10 s intervals, 2 nodes)			
ASCON32	39.2 mW	141.6 h (5.9 d)	Best choice
Salsa20	39.9 mW	139.1 h (5.8 d)	Close alternative
*Mesh network—Dense/high-frequency*			
Dense (1 s intervals, 10 nodes)			
Sosemanuk	79.8 mW	69.5 h (2.9 d)	Best for high-frequency
Salsa20	80.6 mW	68.9 h (2.9 d)	Close alternative
*Bulk transfer—Firmware updates*			
1 MB firmware update			
XChaCha20	11.5 mW	482.6 h (20.1 d)	Fastest transfer
ASCON32	11.5 mW	482.6 h (20.1 d)	AEAD benefit

## Data Availability

Data are contained within the article.
